# Percolation Effect on the Complex Permittivities of Polymer Blends

**DOI:** 10.3390/polym15183751

**Published:** 2023-09-13

**Authors:** Hsien-Wen Chao, Yun-Yu Lai, Tsun-Hsu Chang

**Affiliations:** 1 Department of Physics, National Tsing Hua University, 101, Section 2, Kuang Fu Road, Hsinchu 300044, Taiwan; s9822817@m98.nthu.edu.tw; 2 Department of Polymer Science, University of Akron, Akron, OH 44325, USA; yl125@uakron.edu

**Keywords:** complex permittivity, contour mapping method, enhanced field method, percolation effect, polymer blends

## Abstract

This study focuses on the measurement and analysis of the complex permittivities of polymer blends using the field enhancement method (FEM). The blends, consisting of air-powder or solvent–solute mixtures, are placed in a Teflon holder and inserted into the FEM cavity to determine the complex permittivity. The resonant frequency and quality factor of the FEM cavity coupled with the samples provide information on the blends’ dielectric constant and loss tangents. To extract the complex permittivities of three specific samples of DC-840, MCL-805, and MCL-Siloxane, we employ effective medium theories and the high-frequency structure simulator (HFSS) together with the measured data. The results reveal that when the volume fraction of the DC-840 solute in the xylene solvent surpasses a specific threshold, the dielectric constants and the loss tangents experience a notable increase. This phenomenon, known as percolation, strongly correlates with the viscosity of polymer blends. The observed percolation effect on the dielectric behavior is further elucidated using the generalized dielectric constant and the Debye model. By employing these models, the percolation effect and its impact on the dielectric properties of the blends can be explained.

## 1. Introduction

Integrated circuit (IC) boards and semiconductor technology have become an indispensable part of our daily life. They are employed on various electronic devices, from computers to mobile phones. According to Moore’s law, the number of active devices on one chip will double approximately every two years. However, the density of the ever-increasing transistors on the chip triggers a major concern: power consumption. The microwave power absorbed by dielectric materials per unit volume (W·m^−3^) can be evaluated as [1],

(1) $$P=\alpha CfV^{2}$$

where ω is the angular frequency of the wave, $\varepsilon_{0}$ is the permittivity of free space, $\varepsilon^{\prime }$ is the real part of the complex permittivity, and $\tan\delta =\varepsilon^{\prime \prime }/\varepsilon^{\prime }$ is the ratio of the imaginary part and real part of the complex permittivity. $E_{rms}$ is the root mean square of the electric field (V·m^−1^). To minimize device power consumption, we introduce an insulator of low dielectric constant (Dk, $\varepsilon_{r}=\varepsilon^{\prime }/\varepsilon_{0}$) and low dielectric loss (Df, $\tan\delta =\varepsilon^{\prime \prime }/\varepsilon^{\prime }$) [2,3,4] between the transmission lines. Therefore, developing low-Dk materials with low dielectric loss (Df) is becoming increasingly attractive among chemists, materialists, and engineers [2,3,4,5,6]. The complex permittivity of the composite material can be adjusted by doping dielectric nanoparticles (e.g., Al_2_O_3_, BaTiO_3_, TiO_2_, SiO_2_, ZnO, etc.) or conductive nanoparticles (e.g., silver nanoparticles) into a polymeric host, such as epoxy, polyurethanes, or polyesterimides [7,8,9]. Here, we consider two types of low-Dk materials: organic polymers and inorganic polymers. This article will concentrate on inorganic-type materials, particularly siloxane-based polymers. Because of its longer bond distance, the backbone of the siloxane polymer Si-O-Si bond is more flexible than the C-O-C bond. The bond rotation can vary from 105° to 180° [10]. Consequently, every single Si-O-Si bond can rotate around another [11], resulting in a higher empty volume in the material. The presence of the empty volume leads to a lower dielectric constant because it can occupy more free space whose relative permittivity is nearly one [12]. However, those materials are manufactured in types of powder or gel, leading to difficulty in measuring those materials of complex permittivities. In this article, we propose the field enhancement method (FEM) [13] and the effective medium method [14] to measure the complex permittivities of the materials. We found that when two or more polymers are blended, the electrical property of the blend is significantly different from that of the individual component. This phenomenon is called the percolation effect.

Percolation refers to the phenomenon that occurs in composite materials when there is a significant change in the physical properties of the material as a result of the continuous phase reaching a critical concentration [15,16]. Various factors, including the blend’s morphology, the components’ concentration, the polymers’ nature, and the presence of additives or fillers, influence the percolation behavior and its effect on complex permittivity. Understanding the percolation threshold and its impact on complex permittivity is crucial in designing the desired electrical properties of polymer blends, such as electronic and energy storage applications. The real part of the complex permittivity can dramatically increase or decrease near the percolation threshold. Here, we are interested and will focus on the cases where Dk changes drastically for polymer blends.

This study shows how we characterize three low-dielectric and low-loss materials in powder and gel mixtures. Further, we will provide experimental evidence and explanation that the percolation effect does affect the Dk and Df.

## 2. Sample Preparation and Measurement Procedure

**Figure 1:**
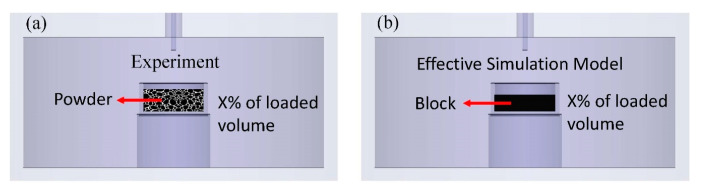
(**a**) The powder was crammed into the Teflon pillbox with suitable pressure. The different percentages of loaded volumes were prepared. (**b**) A modified (effective) model was used in the simulation. The modified model employed a block sample to represent the effects [17].

Figure 1a illustrates the experimental setup, where the powders or gels were placed inside a Teflon pillbox. The capacity of the Teflon’s pillbox was 1.00 cm^3^. The Teflon dielectric constant was around 2.05, and its loss tangent was 0.0001. The contribution of the Teflon pillbox can be calibrated. The samples in the Teflon pillbox were placed in the FEM cavity. The measured port was a 3.5 SMA (Sub-Miniature version A) coaxial connector. From the reflection signal, one can determine the resonant frequencies and Q-factors with different volume fractions. Detailed measurements can be found in Ref. [13]. Then the effective medium (extrapolated) method was employed to evaluate those resonant frequencies and Q-factors in 100% of the loaded volume. Next, those complex permittivities of the samples could be determined. In addition, the powders of the materials were almost impossible to fill into 100% of the materials. Finally, the materials were pressed by simple equipment, and the compression ratio was 70~80% maximum. The higher the compressed rate, the more precise the evaluated complex permittivity.

Three samples were tested. The siloxane resin DC 840 was purchased from Dow Corning Corporation. The other two, MCL-805 and MCL-Siloxane, were synthesized in the Material and Chemical Research Laboratories (MCL), Industrial Technology Research Institute (ITRI). The siloxane resin DC 840 is known for low dielectric constant and loss tangent, while MCL-805 and MCL-Siloxane were synthesized using different techniques. The measured results related to the MCL-805 and MCL-siloxane compounds have never been shown before and are essential for applications. Before the measurement, the typical procedures for preparing DC 840 samples were followed. Remove the residual DC 840 solvent under reduced pressure. Then the product was further dried under 120 °C for 3 h to yield a white powder. All the sample densities were measured by digital density meter (QL-120T). The densities of the powder samples were measured by the pycnometer method in the following step. First, we measured the weight of the solid sample ($m_{s}$) and of the pycnometer filled with liquid ($m_{L}$), respectively. Secondly, we inserted the sample into the pycnometer and then filled it with liquid to measure the total weight of the pycnometer ($m_{s}+{m^{\prime }}_{L}$). Finally, the sample density (*ρ_s_*) could be calculated under the equation, where *V* is the pycnometer volume (constant value) and *V*−*V*_*L*_′ is the sample’s calculated volume.

(2) $$\rho_{S}=\frac{m_{S}}{V-V_{L}^{\prime }}$$

**Figure 2:**
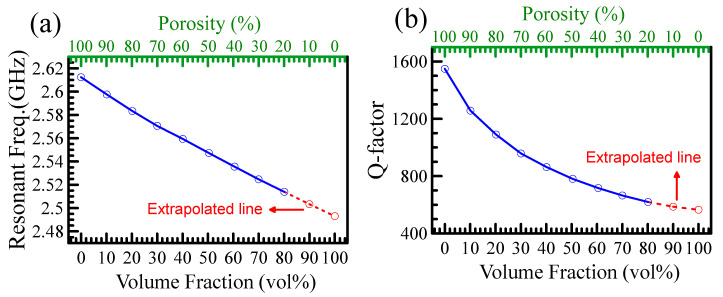
(**a**) shows the measured resonant frequencies versus the volume fraction of the DC 840 powder. The extrapolated line gives the resonant frequency of DC 840 powder at 100% of the loaded volume. (**b**) plots the measured Q-factors as a function of the volume fraction of the DC 840 powder. The Q-factor of the pure DC 840 powder is extrapolated to that of 100%.

The resonant frequencies and Q-factors related to DC 840 volume fraction from 0 to 80% are shown in Figure 2a and Figure 2b, respectively. The extrapolated data to 100% of the loaded volume are depicted in dashed lines. The dielectric constant and loss tangent of powder can be determined by the extrapolated method and the HFSS simulation curve [13,17,18].

**Figure 3:**
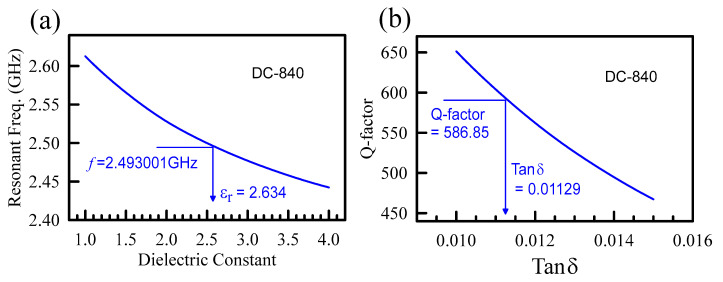
(**a**) shows the relation between the dielectric constant related to the resonant frequency. The curve is plotted with the simulation data of HFSS. Take the DC-840 as an example; the measured resonant frequency is 2.493. Compared with the simulated curve, the calculated dielectric constant is 2.634. (**b**) plots the relation of the loss tangent versus the Q-factor. The measured Q-factor of DC-840 is 586.85, corresponding to a loss tangent of 0.011.

If the loss tangent of the material is low (e.g., tan *δ* ≤ 0.1), the dielectric constant will monotonously depend on the resonant frequency, and the quality factor will depend on the loss tangent, as shown in Figure 3a,b. For a measured resonant frequency of 2.493001 GHz, the corresponding relative dielectric constant is 2.634. Further, for a measured Q-factor of 586.85, the related loss tangent is 0.01129. DC 840 is a medium-to-hard silicone binder resin known for low dielectric constant and loss tangent. The complex permittivities of the three powders are listed in Table 1 with measurement errors. Their dielectric constants are 2.805 and 2.815 for MCL-805 and MCL-Siloxane, respectively. The corresponding loss tangents are 0.005 and 0.017. The lost tangent of MCL-805 is the smallest among the three and is preferred to employ in the insulator of IC board and semiconductor technology.

## 3. Validation of the Characterization

**Figure 4:**
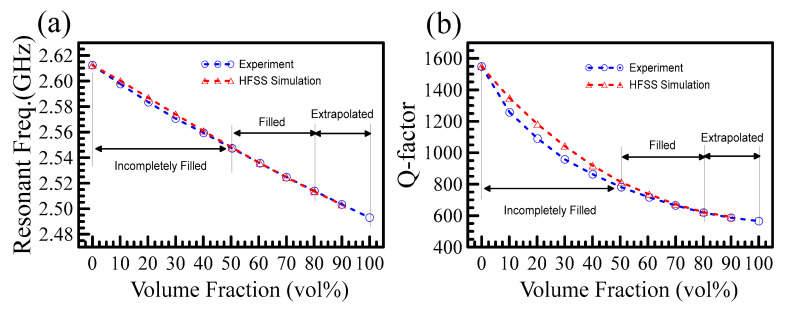
(**a**) shows the relation of resonant frequency related to volume fraction. (**b**) plots the relation of the Q-factor versus the volume fraction. Both figures show a similar trend. When the volume fraction of powder is less than 50%, the experiment and HFSS simulation differ. While the volume fraction of powder is greater than 50%, their results agree very well.

Courtney’s cavity perturbation method is well grounded for complex permittivity measurement [19], but the maximum operating frequency is limited to several GHz due to the size of the cavity and is suitable for low-k material characterization. The enhanced-field method is good for bulk, liquid, fibrous, irregular, and powdery samples. The silicone resin DC-840 is a good example to measure complex permittivity. Figure 4a,b shows that the resonant frequency and the quality factor change with the volume fraction. The circles denote the experimental results, and the triangles represent the simulation using HFSS. The figures can be divided into three regions depending on the volume fraction: incomplete filled, filled, and extrapolated. We aimed to obtain the resonant frequency and quality factor at 100% volume fraction. Experiment and simulation in the incomplete filled region differed as expected, while in the filled region, they agreed well. Based on the excellent agreement in the filled region, we were able to extrapolate the results to 100% volume fraction, which was unattainable in the experiment. Once we obtained the resonant frequency and the Q-factor of DC-840, we were able to evaluate the dielectric constant and the loss tangent using the idea shown in Figure 3.

**Figure 5:**
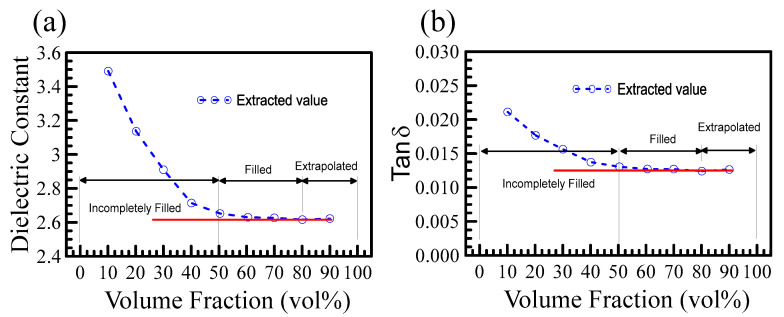
(**a**) shows the extracted dielectric constants versus volume fraction. The dielectric constant is evaluated from the measured resonant frequencies in Figure 4a. (**b**) shows the Q-factors of experimental measurements versus volume fraction. When the volume fraction of the powder exceeds 50%, the extracted dielectric constant and loss tangent agree well with the values extrapolated to 100%.

Figure 5a,b plots the dielectric constant and the loss tangent as functions of the volume fraction based on the measured and simulated data in Figure 4a,b. The figures are divided into three regions: incomplete filled, filled, and extrapolated. Red horizontal lines are drawn to mark the values at 100% volume fraction. The data in the incomplete filled region overestimate, while in the filled region, the extracted values agree well with the extrapolated values at 100%. It is clear from Figure 4 and Figure 5 that the higher the volume fraction, the more accurate the extrapolated values. When the volume fraction was greater than 50%, the obtained dielectric constant and loss tangent agreed well with the extrapolated data at 100%.

## 4. Percolation Effect and Viscosity

In the context of polymer blends, percolation typically refers to the formation of a conductive network within an insulating polymer matrix. Permittivity is a measurement of a material’s ability to store and transmit electrical energy under the influence of an electric field. It consists of two components: the real part (dielectric constant) represents the energy storage capability, and the imaginary part (loss tangent) represents the energy dissipation. The percolation effect can occur when one of the polymer blends forms a continuous phase while the other(s) can form a dispersed phase. As the concentration of the dispersed phase increases, it reaches a critical value at which a conductive pathway is formed throughout the material. This conductive network significantly affects the complex permittivity of the blend. The increase of the dispersed phase is attributed to the formation of conductive paths that allow for enhanced charge transport within the blend. The imaginary part of the complex permittivity, or the loss tangent, can also be influenced by percolation. The loss tangent may decrease near the percolation threshold due to the increased conductivity of the material.

It’s worth noting that the specific behavior of percolation and its effect on complex permittivity can vary depending on the blend composition, processing conditions, and other factors. Experimental characterization and modeling techniques are often employed to study and quantify the percolation phenomena in polymer blends.

**Figure 6:**
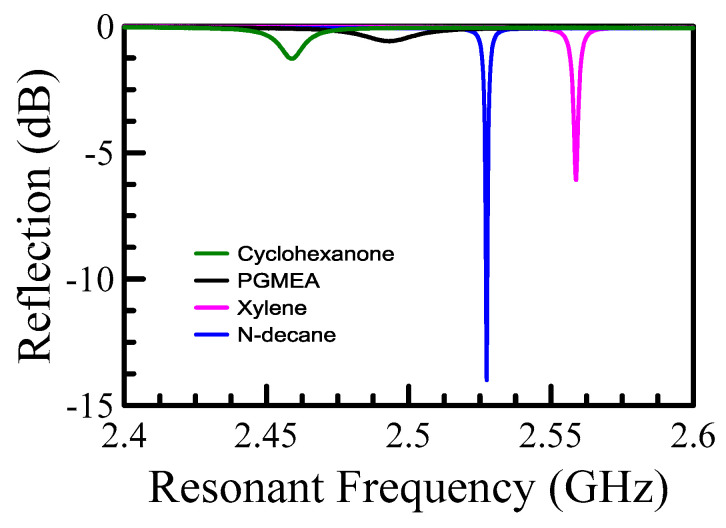
Shows the four solvents, cyclohexanone, PGMEA, Xylene, and N-decane, that can dissolve the DC-840.

**Figure 7:**
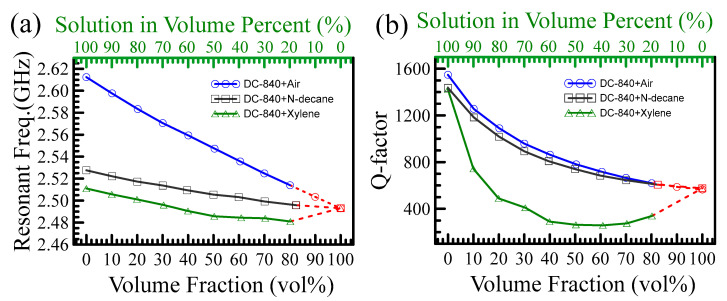
(**a**) shows the relation between resonant frequencies and volume fraction (percentages) for three kinds of solutions. (**b**) shows the relation between Q-factors and volume fraction (percentages) for three kinds of solutions. The red dashed lines are extrapolated curves.

To investigate the percolation effect, we created mixtures of solvent and solute instead of the aforementioned air-powder mixtures. To begin with, we had to choose suitable solvents with a low-loss tangent because a high-loss tangent would dominate the final result. Four different types of solvent could dissolve/partially dissolve DC-840, as displayed in Figure 6. The higher the resonant frequency indicated, the lower the dielectric constant. Further, the higher the Q-factor suggested, the lower the loss tangent. From Figure 6, one can conclude that xylene and N-decane are the preferred solvents. For lossy solvents (i.e., cyclohexanone and PGMEA), the loss tangent is large and will degrade the accuracy and resolution of the measurement. Different volume fraction samples were prepared by stirring and sonication. A similar measurement procedure was introduced as the air-powder model for evaluating resonant frequency. Note that Figure 6 is used to display how we selected suitable solvents (high Q-factor). Accurate resonant frequencies can be found in Figure 7a for 0% volume fraction.

Interestingly, we found drastically different behavior as compared to the air-powder mixture, as shown in Figure 7. The air and N-decane cannot dissolve the DC-840 powder, but the measured curves of the two mixtures (DC-840 + air; DC-840 + N-decane) can be used to find the complex permittivity of DC-840 using the extrapolated method. Although the Xylene can dissolve the DC-840 powder, the curve with open triangles exhibits unexpected behavior called percolation. To further explain this phenomenon, we evaluated DC-840’s intrinsic physical properties, including refractive index and solution viscosity in a dilute solution. The polymer relaxation time relative to the solution viscosity is very high. The higher the viscosity, the higher the relaxation time. In a concentrated solution, polymer motion would confine into a cube. Thus, relaxation time [20,21] is only relative to molecule weight [22,23].

**Figure 8:**
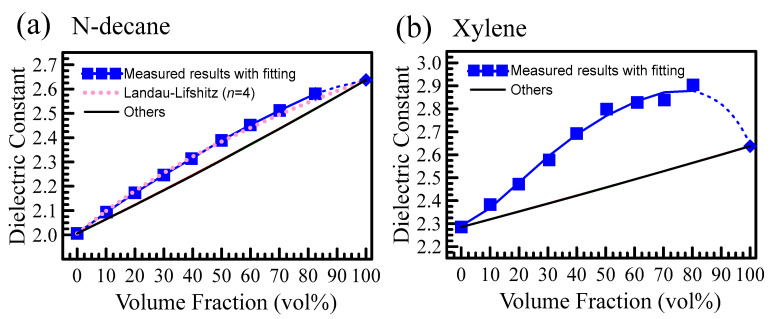
(**a**) shows the relation between the dielectric constant and volume fraction for the solute of DC-840 and solvent of n-decane: measured results (blue squares) and the fitting (lines). The LLL model with n=4 agrees well with the experiment. Others, including Maxwell-Garnet, LLL with n=1/3, Brugeman, and PvS models, predict the same trend (black line). (**b**) displays the relation between the dielectric constant and volume fraction for the solute of DC-840 and solvent of Xylene: measured results (blue squares) and the fitting (lines). Other models, including Maxwell-Garnet, LLL with n=1/3, Brugeman, and PvS models, predict the same trend (black line), but all the effective medium theories are unable to fit the experimental results.

Effective medium theories are handy in estimating the effective permittivity of composites ($\varepsilon_{eff}$). Maxell-Garnet, Bruggeman, and PvS models can be found in Ref. [24]. Lichtenecker’s law [14] is found to be helpful in the current study. It reads,

(3) $$\varepsilon_{eff}^{n}=v_{p}\varepsilon_{p}^{n}+v_{h}\varepsilon_{h}^{n}$$

where $\varepsilon_{p}$ is the permittivity of the powders (i.e., DC-840) and $\varepsilon_{h}$ is the permittivity of the host matrix (air/n-decane/Xylene). The volume fraction of the particle (host) is denoted as $v_{p}$ ($v_{h}=1-v_{p}$). The value n is to be determined. n=1/3 is the so-called Landau-Lifshitz-Looyenga (LLL) model. The LLL model can be applied to mixtures with irregularly shaped dopants, such as porous materials. Interestingly, the abovementioned four effective medium theories predict the same behaviors as shown in Figure 8a,b. The predictions from the four models overlap. Thus, we introduce a new LLL model with n=4 for the solvent of n-decane (See Figure 8a). However, the physical reason(s) for such a high value of n=4 requires further investigation. For the solvent of Xylene (Figure 8b), we cannot find a suitable effective medium theory. Therefore, the generalized dielectric constant and Debye relaxation model will be introduced to explain the bizarre experimental findings (i.e., the percolation effect).

The generalized dielectric constant is shown below.

(4) $$\varepsilon (\omega )=1+\frac{e^{2}}{m}\sum_{j(\mathrm{bound})}\frac{Nf_{j}}{\left(\omega_{j}^{2}-\omega^{2}-i\omega \gamma_{j}\right)}+i\frac{e^{2}Nf_{0}}{m\omega (\gamma_{0}-i\omega )}$$

where $Nf_{j}$ is the number of bound electrons of the *j*-th kind per unit volume and $Nf_{0}$ is the number of free electrons per unit volume. ω is the driving frequency of the wave. $\omega_{j}$ and $\gamma_{j}$ are the natural resonant frequency and the collision frequency of the *j*-th kind bound electrons, respectively. Equation (4) considers the contribution from the bound and the free charges. For the case of non-zero free electrons, i.e., $Nf_{0}\neq 0$, Equation (4) can be rewritten as:

(5) $$\varepsilon (\omega )=\varepsilon_{b}^{\prime }+i\varepsilon_{b}^{\prime \prime }+i\frac{\sigma }{\omega }$$

where $\sigma =e^{2}Nf_{0}/m\gamma_{0}$ with the condition $\gamma_{0}\gg \omega$. Equation (5) tells that,

(6) $$\tan\delta =\frac{\varepsilon_{b}^{\prime \prime }+\frac{\sigma }{\omega }}{\varepsilon_{b}^{\prime }}$$

The DC-840 solute can dissolve in the xylene solvent, and the solution possesses free ions (σ≫0). The free charges might partially explain why the loss tangent of the blend increases, as in Figure 8a,b. However, the generalized dielectric constant cannot fully explain the behavior observed in Figure 8b, where the mixture’s dielectric constant is greater than that of the pure substance (i.e., DC-840 and Xylene). Here, we introduce the Debye relaxation model to explain the observed phenomena. The Debye model is a classical dielectric model used to explain the dielectric behavior of materials, including polymer blends. The complex permittivity of the polymer blend can be calculated as the individual relaxation process using the Debye model equation [25]:

(7) $$\varepsilon^{\prime }\left(\omega \right)+i\varepsilon^{\prime \prime }\left(\omega \right)=\varepsilon_{\infty }+\frac{\varepsilon_{s}-\varepsilon_{\infty }}{1+i\omega \tau }$$

where $\varepsilon_{s}$ ($\varepsilon_{\infty }$) is the material’s permittivity at “static” (“infinite frequency”) frequency, ω is the angular frequency, and τ describes a relaxation time. In nonpolar dielectrics, a complex permittivity has only a real part and is frequency-independent. In polar dielectrics, the polymer blend consists of multiple relaxation processes (relaxation times τ). These times correspond to a time of stabilization or demise of the polarization state after switching on/off an external electric field. We should sum up all the contributions. At the percolation threshold, where the blend changes from a liquid state to a gel-like state, the relaxation time (τ) changes. The generalized dielectric constant (Equation (4)) and the Debye relaxation model (Equation (7)) just qualitatively and partly explain the observed effect in Figure 8. Detailed investigation is needed to provide a more in-depth quantitative study.

**Figure 9:**
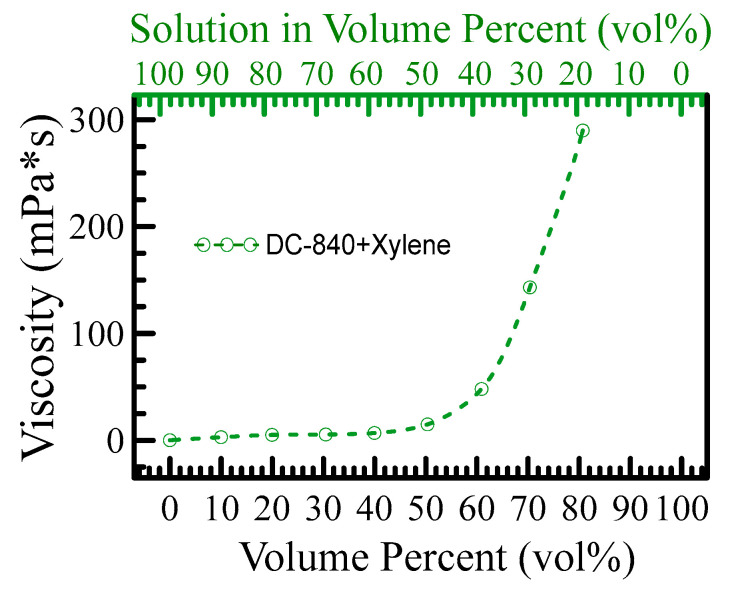
Shows the viscosity of a mixture versus the percentage of solute. When the percentage of solute exceeds 50%, the molecules of DC-840 are entangled with each other. The percentage of DC-840 gets increasingly higher, and the behavior of a mixture is similar to a solid state. When the percentage of solute is lower than 50%, the molecules of DC-840 have enough space to extend their chains. They hardly contribute to the viscosity of the blend.

Figure 9 shows the measured viscosity versus the volume percent. The viscosity was measured by DV-II+Pro Viscometer, which could measure within the range of 100–40,000,000 mPa*s (cP) by appropriated spindle selection. All the results lay in the torque range of 10–100. The measurement of density is critical. However, it is hard for the powder to reach the compression of 100%, and one cannot determine its volume. In other words, the volume of the powder is difficult to determine, especially since this material can be dissolved in a solvent. It must be calculated for its density and mass, so it is a linchpin in the accurate evaluation of its density.

Figure 9 reveals a dramatic increase in the measured viscosity when the solute contains more than 50%. The viscosity could reach 289.8 mPa*s in the maximum solution of 80.7 vol%. When the DC-840 contains more than 80.7 vol%, the DC-840 powder cannot completely dissolve in the solvent of Xylene. In addition, it is fascinating and unusual that the dielectric constant of a mixture with solute percentage (DC-840) from 40 to 100% is higher than that of DC-840 and Xylene, shown in Figure 8a. For application, the dielectric constant of the mixture is controlled by tuning the solute percentage. When the solute contains more than 50%, the viscosity grows very fast because the molecular chains are entangled with each other. When the molecular chain is reduced, the relaxation time is shortened, and the dielectric constant of the blend is increased. The solute percentage gets increasingly higher, and the parts of the molecular chain are more and more easily entangled with each other, leading to an increase in the relaxation time of the blend.

The percolation effect in polymer blends can impact the viscosity of the mixture [26,27,28,29,30]. When a percolation network forms within a polymer blend, it introduces connectivity among the dispersed phase particles or fillers. This connectivity can affect the flow behavior and viscosity of the mixture in several ways: 1. non-Newtonian behavior, 2. enhanced viscosity, and 3. solid-like behavior. In some cases, the percolation effect can lead to a transition from a liquid-like to a gel-like or solid-like behavior. This transition results in a significant increase in the viscosity, leading to the blend behaving more like a solid than a liquid.

It’s worth noting that the relation between the percolation effect and the viscosity of a polymer blend can be complex and highly dependent on various factors, including the type of the polymers, the concentration and morphology of the dispersed phase, and the presence of additives. The presence of percolation can introduce changes in the flow behavior, viscoelastic properties, and rheological characteristics of the mixture.

Rheological measurements are commonly employed to characterize the viscosity and flow behavior of polymer blends near the percolation threshold. The proposed complex permittivity measurement is shown to be a new and valuable means to study the relation between the percolation effect and the viscosity of the mixture in different blend systems.

## 5. Conclusions

In conclusion, this study investigated the complex permittivity of air-powder and solvent–solute blends using a FEM and explored the percolation effect on the dielectric behavior. By measuring the resonant frequency and quality factor of the FEM cavity with the samples, the dielectric constant and loss tangent were determined, providing valuable insights into the electrical properties of the blends. The measured resonant frequencies and quality factors can be used to extract the complex permittivities of three specific samples: DC-840, MCL-805, and MCL-Siloxane. Notably, the volume fraction of the DC-840 solute in the xylene solvent played a crucial role in the observed percolation effect. When this volume fraction exceeded a certain threshold, both the dielectric constant and the loss tangent exhibited a significant increase. This finding highlighted the existence of percolation in the blends and demonstrated a strong correlation between the percolation effect and the viscosity of the polymer blend.

The generalized dielectric constant and the Debye models were employed to explain the percolation effect on the dielectric behavior. These models provided a theoretical framework for understanding the observed changes in the electrical properties near the percolation threshold. This study contributes to the understanding of complex permittivity in polymer blends and sheds light on the percolation phenomenon and its correlation with viscosity. The findings have implications for the design and optimization of polymer blend systems, where control over the percolation threshold can lead to tailored electrical properties and enhanced performance in various applications such as electronic devices, sensors, and energy storage systems. Further theoretical and experimental investigations can delve into the specific mechanisms underlying the percolation effect and explore additional factors, such as the particle-size effect and the interfacial effect.

## Figures and Tables

**Table 1:** Shows the measured dielectric constant and the loss tangent of the three powders, DC-840, MCL-805, and MCL-Siloxane.

	DC-840	MCL-805	MCL-Siloxane
Dielectric Constant ($\varepsilon_{r}=\varepsilon^{\prime }/\varepsilon_{0}$)	2.634 ± 0.000105	2.805 ± 0.000186	2.815 ± 0.000127
Loss Tangent ($\tan\delta =\varepsilon^{\prime \prime }/\varepsilon^{\prime }$)	0.01185 ± 8.38 × 10^−5^	0.005 ± 8.42 × 10^−5^	0.017 ± 4.24 × 10^−5^

## Data Availability

The data presented in this study are available on request from the corresponding author.
